# Post-stroke butyrate treatment shows sex-dependent microglial responses but does not improve outcomes in a mouse model of endothelin-1 sensory motor stroke

**DOI:** 10.1186/s12868-025-00959-3

**Published:** 2025-07-17

**Authors:** Ashley de Witte, Juliana Montoya Sanchez, Emerson Daniele, Jingan Chen, Yibang Fan, Pranav Khatri, Daniela Lozano Casasbuenas, Angel Zhang, Kathryn G. Todd, Maryam Faiz, Matthew Churchward

**Affiliations:** 1 Division of Anatomy, Department of Surgery, University of Toronto, Toronto, ON Canada; 2 Neurochemical Research Unit, Department of Psychiatry, University of Alberta, Edmonton, AB Canada; 3 Institute of Medical Sciences, University of Toronto, Toronto, ON Canada; 4 Department of Laboratory Medicine and Pathobiology, University of Toronto, Toronto, ON Canada; 5 Department of Biological Sciences, Concordia University of Edmonton, Edmonton, AB Canada

**Keywords:** Butyrate, Short-chain carboxylic acids (SCCAs), Sex differences, Ischemic stroke, Microglia, Gut-brain axis, Short-chain fatty acids (SCFAs), Stroke

## Abstract

**Background:**

Stroke induces gut dysbiosis and reduces microbial production of short-chain carboxylic acids (SCCAs), which negatively correlates with stroke outcomes. Previous studies have demonstrated that SCCA supplementation can improve functional recovery, with one recent study suggesting this occurs via modulation of microglial responses. However, the effects of individual SCCAs on microglial responses remain unclear, particularly across sexes and following a more clinically relevant, post-stroke treatment protocol. To address this gap, we investigated the effect of post-stroke supplementation with butyrate on stroke outcomes and microglial responses in both male and female mice over time.

**Results:**

Post-stroke butyrate treatment produced sex-specific microglial responses. In females, butyrate increased microglial ramification at chronic timepoints in vivo and enhanced IL6 release following IFNγ stimulation in vitro. These microglial changes were not observed in males. Despite the distinct microglial responses, butyrate treatment did not correlate with improved stroke outcomes in either sex, as measured by lesion volume and functional recovery.

**Conclusions:**

Our findings reveal previously unknown sex differences in microglial responses to butyrate following stroke. Despite these microglial changes in females, butyrate treatment did not improve functional outcomes in either sex, suggesting that sex-specific optimization of dosing and delivery may be needed for therapeutic efficacy.

**Supplementary Information:**

The online version contains supplementary material available at 10.1186/s12868-025-00959-3.

## Background

Ischemic stroke, caused by occlusion of a cerebral artery, leads to decreased blood supply and oxygenation that results in neuronal death and neurological deficits [1, 2]. Beyond its direct neurological impacts, stroke induces significant alterations in gut microbiota composition, termed gut dysbiosis [3]. This dysbiosis influences the microbial production of metabolites, including short-chain carboxylic acids (SCCAs) [4, 5], which are by-products of microbial dietary fibre fermentation. SCCAs, including acetate, propionate, and butyrate, can readily cross the blood-brain barrier to affect brain function in both health and disease [6–8]. Notably, in stroke patients, increased SCCA levels negatively correlate with severity, neurological deficits, and infarct volume, with butyrate showing the strongest negative correlation [9].

These observations have led to exploration of supplementation with exogenous SCCAs as a therapeutic approach. A seminal study by Sadler et al. [6], demonstrated that pre-stroke supplementation, beginning 4 weeks prior to stroke, with a combination of SCCAs improved post-stroke recovery in male mice [6]. The study identified microglial modulation as a potential mechanism, suggesting that SCCAs could prime these immune cells before stroke or alter post-stroke pathophysiology [6]. However, the pre-stroke treatment paradigm and use of combined SCCAs limit the understanding of the therapeutic potential and mechanism of individual SCCAs in a clinically relevant post-stroke treatment paradigm.

As the immune cells of the central nervous system, microglia respond to invading pathogens, foreign bodies, and injury [10]. Their activation and reactivity are heterogeneous and temporally dynamic, with different activation pathways leading to diverse phenotypes [11–13]. These phenotypes are associated with various changes in morphology, molecular profiles, and function that significantly impact stroke outcomes [11, 12]. During ischemic stroke, microglia produce pro-inflammatory cytokines such as tumor necrosis factor (TNF), interleukin 1 beta (IL1β), interleukin 6 (IL6), and proteolytic enzymes such as matrix metalloproteinase 9 (MMP9) [14, 15]. Previous studies have shown that SCCA treatment can decrease pro-inflammatory cytokine release in microglia, with evidence supporting effects of both butyrate alone and combined SCCA treatment [16, 17].

Despite growing evidence for SCCAs as potential stroke therapeutics [6, 9, 18], with butyrate as the most promising candidate [9, 18], significant knowledge gaps remain. Few studies have focused on the effect of butyrate treatment post-stroke, and how it impacts cellular responses in the CNS. Moreover, sex differences have been largely ignored, with no studies investigating the differential effects of butyrate across sex [6, 9, 18, 19]. Therefore, this study aimed to determine whether post-stroke butyrate treatment is sufficient to improve functional recovery in both males and females and how it changes the microglial response to injury over time. While we observed sex-differences in microglial morphology, and cytokine release, the only sex-specific response to butyrate treatment was an increase in microglial ramification in chronic stroke and an increase in IL6 in response to IFNγ stimulation in females. Nonetheless, these microglial effects were not correlated with improved stroke outcomes as measured by lesion volume and functional recovery.

## Methods

### Animals

*Animal ethics statement* All animal research was conducted under approved Animal Use Protocols from the University of Toronto (Protocol: 20012650) and the University of Alberta Animal Care Committees, in accordance with Canadian Council for Animal Care guidelines.

*In vivo studies* 8- to 10-week-old C57BL6 males and females were purchased from Charles River (Montreal, Quebec, Canada) and housed 4/cage in Allentown cages (length = 29 cm, width = 18 cm, height = 12 cm) in a temperature-controlled room with a 12:12 light-dark cycle. Each cage contained corncob bedding, nesting material, and a climbing structure. Animals received standard rodent chow (18% protein; Envigo, 2918) and non-acidified water ad libitum.

*Gut microbiota normalization* Every other day during the 2-week acclimation period, 100 mL of soiled bedding was removed from each cage, mixed, and redistributed across all cages to normalize starting gut microbiota.

*In vitro studies* Primary microglia were isolated from postnatal day 2 CX3CR1(+/eGFP) mice (*n* = 14 pups).

### Endothelin 1 (ET-1) stroke

ET-1 stroke was performed as previously described in Daniele et al. [20]. Briefly, mice were anesthetized with inhaled isoflurane and 1 µl of ET-1 was stereotaxically injected into three sites in the sensory motor cortex (site 1: + 2 mm ML, + 0.0 AP, + 1.2 DV. Site 2: + 2 mm ML, + 1.0 AP, + 1.2 DV. Site 3: + 2 mm ML, + 2.0 AP, + 1.2 mm DV). For sham injured controls, bore holes were drilled at matching coordinates without ET-1 injection. Analgesia was provided via meloxicam (SC, 5 mg/kg) intra-operatively and for two days post-surgery.

### Sodium butyrate administration

Post-surgery, mice received sodium butyrate (40 mM; Sigma Aldrich, 303410-100G) in their drinking water *ad libitum* until sacrifice. Vehicle-treated controls received sodium chloride (40 mM; BioShop, SOD002.205).

### Behavioural testing

All behavioural testing was performed during the light-phase of the light-dark cycle and animals were allowed to habituate the testing room for 30 min prior to testing.

### Cylinder task

The cylinder task was used to assess paw dragging behaviour, as previously described in Daniele et al. [20]. After acclimatization to the testing room, animals were removed from the home cage and placed into a plexiglass cylinder positioned on a mirrored platform. Fifteen rears were recorded per animal. A blinded experimenter analyzed the videos and counted the number of paw touches and paw drags. Animals with no initial deficit or with less than 10 total touches with the left or right paw were excluded from the study. Paw drag percentage was calculated as: (# of affected paw drags/(# of affected paw touches + # of affected paw drags)) × 100. Changes in paw dragging were calculated relative to baseline to account for natural variation: Δpaw drag = (paw drag _time 2_ − paw drag_D0_). *n* = 20 animals were removed from the study.

### Ladder task

Ladder testing was performed to assess functional deficits following stroke and was based on the protocol previously described by Metz & Whishaw [21]. Ladder rungs were set up at an inter-rung distance of 0.5 cm to 1.5 cm. An empty basin was placed at one end of the ladder and a dark goal box at the other end of the ladder. Animals were acclimatized to the testing room for 30 min prior to training and testing sessions. Training was performed once a day for two days prior to baseline testing. For training, each animal was removed from the home cage and placed at the start of the ladder apparatus and was allowed to cross to the goal box. The animal was allowed to rest in the goal box for 30 s–1 min before the second habituation trial. Animals that did not acquire the task following two days of training were excluded from the study. For testing, a GoPro camera was placed underneath the ladder so the entire length of the ladder apparatus was in frame. After beginning the recording, the animal was removed from their home cage at the end of the ladder and placed directly onto the beginning of the ladder apparatus. Animals were allowed to rest for 30 s–1 min in the goal box. This was repeated for four trials. Testing was performed one week prior to surgery (baseline; D0), and 4 days after stroke (D4). A blinded experimenter analyzed the videos and counted the number of steps and slips for each limb (left and right, hindlimb and forelimb). The % slippage was calculated using the following formula: ((# left limb slips - # right limb slips)/(# of total steps)) × 100, averaged across the 4 trials.

### Animal euthanasia

At day 4 (D4) or 20 (D20) post-stroke, animals were anesthetized with an overdose of Avertin (250 mg/kg, I.P.) and subsequently underwent intracardiac perfusion with 30 mL of PBS (Phosphate-Buffered Saline) followed by 30 mL of 4% paraformaldehyde. Mice were decapitated, and brains were harvested for downstream tissue processing.

### Tissue processing

Brains were post-fixed in 4% PFA overnight, then transferred to 30% sucrose solution for cryoprotection. Coronal serial Sect. (20 μm) were cut using a ThermoScientific HM525 NX cryostat onto SuperFrost slides (Fisherbrand, 1255015) from AP + 2.0 mm to AP − 1.0 mm.

### Immunohistochemistry

Slides were washed three times in PBS. Tissue sections were permeabilized for 15 min in 0.5% triton solution and blocked (10% normal goat serum, 0.5% bovine serum albumin, 0.5% triton) for 1 h at room temperature. Sections were incubated in primary antibodies (Table 1) diluted in blocking buffer at 4 °C. Following three washes in PBS, sections were incubated with appropriate secondary antibodies diluted in blocking buffer for 1 h at room temperature. Slides were washed three times in PBS, counterstained in DAPI solution (1:1000 in PBS) for 5 min, washed three times in PBS, and mounted using Mowiol (SigmaAldrich, Cat #: 81381).

**Table 1:** IHC reagents

Reagent	Dilution	Company, Catalogue #
Rabbit anti-IBA1	1:200	Wako, 019-19741
Rabbit anti-NEUN	1:200	Sigma-Aldrich, ABN78
Goat anti-Rabbit Alexa 568	1:400	Invitrogen, A11036
DAPI	1:3000	Invitrogen, S33025

### IBA1+/DAPI + image acquisition

Confocal z-stack images (1 μm apart) were acquired with a Zeiss LSM 900 confocal microscope using Zen Blue software (Zeiss, Germany). Image threshold intensity was set based on background signal measured in secondary only controls. Linear brightness adjustments were made to all channels in Zen software. Guided by 10x reference images, two 40x images were obtained lateral and medial to the core of the infarct in three coronal sections (AP + 0.0 mm, AP + 1.0 mm and AP + 2.0 mm). Individual images from the z-stack were then merged using Fiji/ImageJ software [22].

### Sholl analysis

Using the ImageJ software, individual cells were cropped from 40x confocal images then preprocessed by binarization, background removal, and skeletonization. Sholl analysis of morphology [23–25] was then performed on preprocessed images using the SNT, Sholl Analysis plugin on ImageJ. Analysis parameters were set so the start radius was the edge of the cell soma, and the step size was 1 μm. The end radius was adjusted to ensure full coverage of each cell, and the number of primary branches was manually counted and inputted into the plugin. The number of primary branches, the total number of intersections, the maximum number of intersections, the radius at which the maximum intersections occurred, the ramification index, and the enclosing radius (radius of the farthest intersection) were measured. For acute stroke (D4), 89 IBA1^+^ cells were analyzed from 3 stroke-injured vehicle-treated males, 136 IBA1 + cells were analyzed from 5 stroke-injured butyrate-treated males, 111 IBA1 + cells were analyzed from 4 stroke-injured vehicle-treated females, 164 cells were analyzed from 5 stroke-injured butyrate-treated females. For chronic stroke (D20), 131 IBA1 + cells were analyzed from 5 stroke-injured vehicle-treated males, 87 IBA1 + cells were analyzed from 4 stroke-injured butyrate-treated males, 113 IBA1 + cells were analyzed from 5 stroke-injured vehicle-treated females, 113 cells were analyzed from 5 stroke-injured butyrate-treated females. All Sholl metrics were averaged per animal for downstream statistical analysis.

### Cell counts

In the same images used for Sholl analysis, DAPI+/IBA1 + cells were counted using ImageJ analysis software [26] with the points tool. Cell counts were expressed as the proportion of IBA1 + DAPI + cells over DAPI + cells per field of view (159.73 μm x 159.73 μm).

### Lesion volume analysis

NEUN/DAPI stained sections from AP + 2.0 to AP − 1.0 mm were imaged using a Zeiss Axioscan slide scanner. Lesion volume was defined as the region devoid of NEUN staining. Using QuPath software [27], lesion area was measured in 20 μm thick coronal Sect. (160–180 μm apart) spanning the anterior–posterior extent of the injury site. Total infarct volume (V) was calculated as:

$$\:V={\sum\:}_{i=1}^{n}\left(\frac{{A}_{i}+{A}_{i+1}}{2}\right)D$$

where A = lesion area of section i, and D = distance between sections.

### Cell culture reagents

Cell culture media components (DMEM F12, FBS, penicillin/streptomycin, 0.25% trypsin/EDTA, HBSS) were from Gibco (Thermo-Fisher Scientific, Ottawa, ON). Additional reagents included: *E. coli* O111:B4 LPS, poly-l-lysine (PLL), and butyrate (Millipore Sigma, St. Louis, MO); IFNγ (Peprotech, Montreal, QC); and ELISA kits for TNF, IL1β, IL6, and IL10 (R&D systems, Minneapolis, MN).

### Primary microglial cell culture

Microglia were isolated from CX3CR1^(+/eGFP)^ postnatal day 2 (PN2) mice. A total of 14 mouse pups were used for these experiments. Pups were visually identified as either male or female by a laboratory technician not involved in experimental work to keep the experimenter carrying out cell culture growth and treatment blinded to sex. Following decapitation, brains were extracted, and meninges removed in warm HBSS (37 °C). Single-cell suspensions were generated by enzymatic dissociation in 0.25% trypsin/EDTA (37 °C) for 20 min followed by mechanical trituration with a serological pipette. Cells were washed in DMEM-F12, 10% FBS, 200 U/ml penicillin, and 200 ug/ml streptomycin, centrifuged, and mechanically triturated again. The cells were then plated in 12 well plates coated with PLL (1 brain per plate) and cultured with DMEM/F12, 10% FBS, 200 U/ml penicillin, and 200 ug/ml streptomycin for 14 days in a humidified 5% CO2 incubator until confluent. At 14 days, microglia were isolated by treatment with 0.25% trypsin/EDTA diluted 1:3 with DMEM/F12 for 15 min to remove astrocytes and oligodendrocytes. Adherent microglia were rinsed with DMEM/F12, 10% FBS, 200 U/ml penicillin, 200 ug/ml streptomycin and subsequently maintained in DMEM/F12 for 24 h until experiments [28]. Cell culture purity was routinely analyzed using fluorescent microscopy. CX3CR1^(+/eGFP)^ fluorescent microglia were analyzed in contrast with the nuclei stain Hoechst and experiments were only used if microglia purity was ≥ 95%.

### Primary cell culture butyrate treatments

**Fig. 1:**
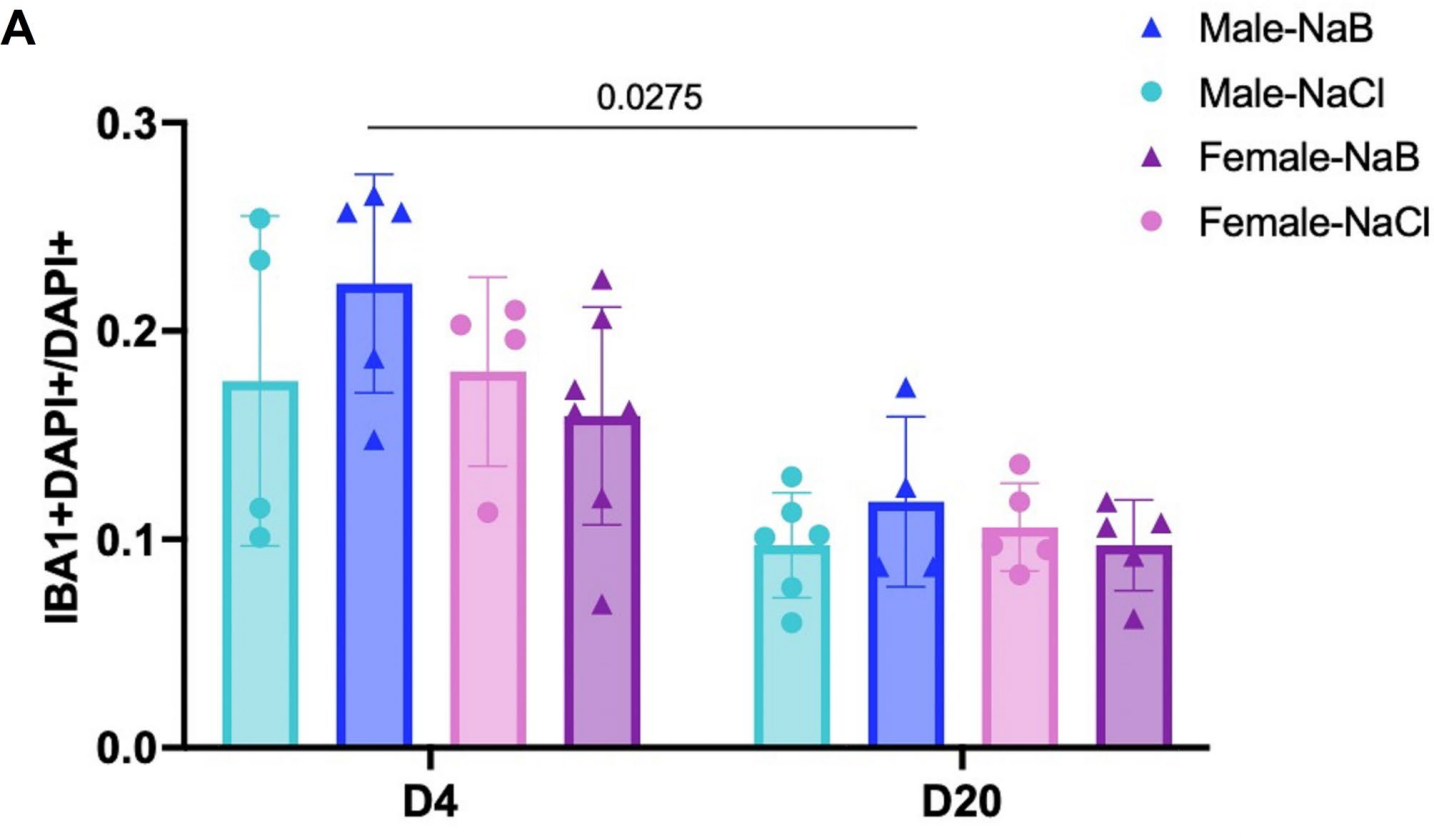
Butyrate treatment does not alter microglia number. (**A**) Microglia number was counted in acute (D4) and chronic (D20) stroke. Counts are represented as the number of IBA1- and DAPI-positive cells per DAPI-positive cells. Cells were counted both medially and laterally to the lesion. Counts were performed on both male (blue) and female (pink/purple) animals that were treated with either butyrate (NaB; dark blue/purple) or vehicle (NaCl; light blue/pink). 3-way ANOVA with Tukey’s multiple comparisons test. Significant p values (< 0.05) are represented on graphs. ND4−male−NaCl = 4 animals; ND4−male−NaB = 5 animals; ND4−female−NaCl = 4 animals; ND4−female−NaB = 6 animals; ND20−male−NaCl = 6 animals, ND20−male−NaB = 4 animals; ND20−female−NaCl = 5 animals; ND20−female−NaB = 5 animals

Microglia were treated with butyrate (0µM, 40µM, 200µM, and 1000µM) for 24 h (Fig. 1), as previously described by Churchward et al. [17]. Butyrate concentrations for cell culture treatment were determined in a previous study [17] and correspond to the measured range of butyrate in rodent brain tissue after gut microbiota manipulation [29]. After 30 min, cells were stimulated with LPS (100 ng/ml) or IFNγ (100 ng/ml). After 24 h, cell culture media was collected for cytokine analysis (TNF, IL1β, IL6, and IL10) using an enzyme-linked immunosorbent assay (ELISA). Microglia were fixed with 5% formalin (5 min) and cell counts were determined using a SpectraMax i3x Imaging Cytometer.

### Statistical analysis

All data were tested for normality using the Shapiro-Wilk test. For nonparametric data, appropriate nonparametric statistical tests were used. All data were plotted and analyzed using Graphpad Prism Version 9.4.0 for Mac OS X, Graphpad Software, San Diego, California, USA. Microglia number and lesion volume data were analyzed with a three-way ANOVA and Tukey’s multiple comparison *post hoc*. Graphs are presented as the mean ± SEM. Microglial morphology was analyzed by averaging the values per animal and performing a two-way ANOVA with uncorrected Fisher’s LSD *post hoc*. Graphs are presented as the mean ± SEM with each dot representing a cell and each colour hue representing an animal. Cell culture data was analyzed with a two-way ANOVA and Sidak’s multiple comparison *post hoc*. Each cell culture experiment was prepared from a single male or female PN2 mouse brain and each was replicated 7 times for a total of 14 independent cell culture experiments. Graphs are presented as the mean ± SEM and each *N* represents the numbers of each experiment conducted using a separate primary cell culture isolated from a separate mouse brain. Behavioural data was analyzed with a two-way ANOVA and uncorrected Fisher’s LSD *post hoc*. Only the significance of within-group across-time comparisons and across-group within-time comparisons was reported for all experiments except cell culture. Graphs are presented as the mean ± SEM. A p-value of ≤ 0.05 was considered significant for all experiments.

## Results

*Post-stroke butyrate supplementation differentially affects microglial morphology across sex and time.*

To understand how post-stroke butyrate supplementation affects microglial responses, we examined microglial number and morphology in male and female mice following endothelin-1-induced stroke at D4 and D20, representative of acute and chronic stroke.

Quantification of IBA1 + cells in the peri-infarct region revealed no differences in microglial number between butyrate-treated and vehicle-treated male and female animals at D4 (Fig. 1) or D20 (Fig. 1). When we examined how the number of microglia changed over time, we found a decrease in IBA1 + cells in butyrate-treated males at D20 compared to D4 (Fig. 1), but no difference in females (Fig. 1).

**Fig. 2:**
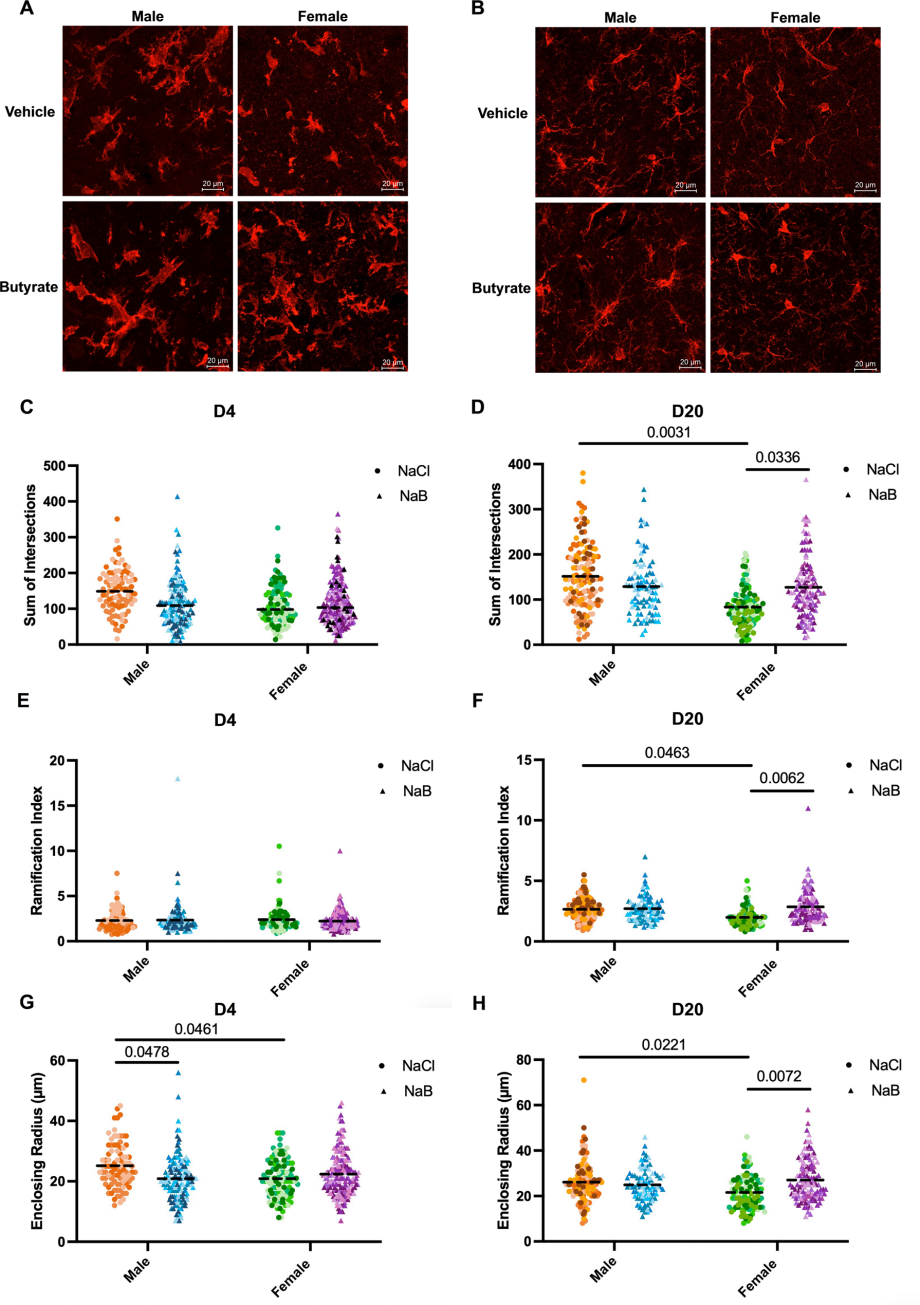
Butyrate treatment alters microglial morphology during chronic stroke in a sex-dependent manner. Representative 40x images of IBA1 + microglia during acute (**A**) and chronic (**B**) stroke. The sum of intersections was measured in acute (D4) (**C**) and chronic (D20) (**D**) stroke. The ramification index was calculated in acute (**E**) and chronic (**F**) stroke. The enclosing radius was measured in acute (**G**) and chronic (**H**) stroke. All measurements were performed on both male and female animals, in addition to both butyrate-treated (blue and purple) and vehicle-treated (orange and green) animals. Each dot represents a cell. Each colour represents an animal. The horizontal dashed line represents the mean of the data. 2-way ANOVA with uncorrected Fisher’s LSD was performed using the average cell morphology metrics per animal, significant p values (< 0.05) are represented on graphs. N_D4−male−NaCl_ = 3 animals, 89 cells; N_D4−male−NaB_ = 5 animals, 136 cells; N_D4−female−NaCl_ = 4 animals, 111 cells; N_D4−female−NaB_ = 6 animals, 186 cells; N_D20−male−NaCl_ = 5 animals, 131 cells, N_D20−male−NaB_ = 4 animals, 87 cells; N_D20−female−NaCl_ = 5 animals, 113 cells; N_D20−female−NaB_ = 5 animals, 117 cells.

Next, we examined microglial morphology using Sholl analysis on skeletonized images of individual IBA1 + microglia at D4 and D20 and averaged morphology metrics per animal (Fig. 2A, B, Additional file 1). At D4, no differences in the total intersections (Fig. 2C), ramification index (Fig. 2E), number of primary branches (Fig S1A), maximum number of intersections (Fig S1B), or the maximum intersection radius (Figure S1E) were observed between males and females. However, female microglia showed a decrease in enclosing radius, suggesting smaller cell size (Fig. 2G) at this acute time point. Butyrate treatment did not alter any of these morphological parameters in females (Fig. 2C, E, G, Figure S1A, C,E). Whereas butyrate-treated males showed a decrease in enclosing radius compared to vehicle-treated controls (Fig. 2G).

In contrast, at D20, we observed marked sex differences in microglial morphology across metrics (Fig. 2D, F, H Fig S1D, F). Females displayed less ramified microglia compared to males, as evidenced by a decrease in total intersections, ramification index, maximum number of intersections, and the maximum intersection radius (Fig. 2D, F, H and Fig S1D, F). As observed at D4, female microglia showed a decrease in enclosing radius (Fig. 2H). Notably, butyrate treatment increased microglial ramification in females evidenced by an increase in total intersections, ramification index, and the maximum intersection radius (Fig. 2D, F, H and Fig S1F). This treatment effect was not observed in males, suggesting a sex-specific response to butyrate in chronic stroke.

### Sex-dependent microglial cytokine responses to butyrate treatment

**Fig. 3:**
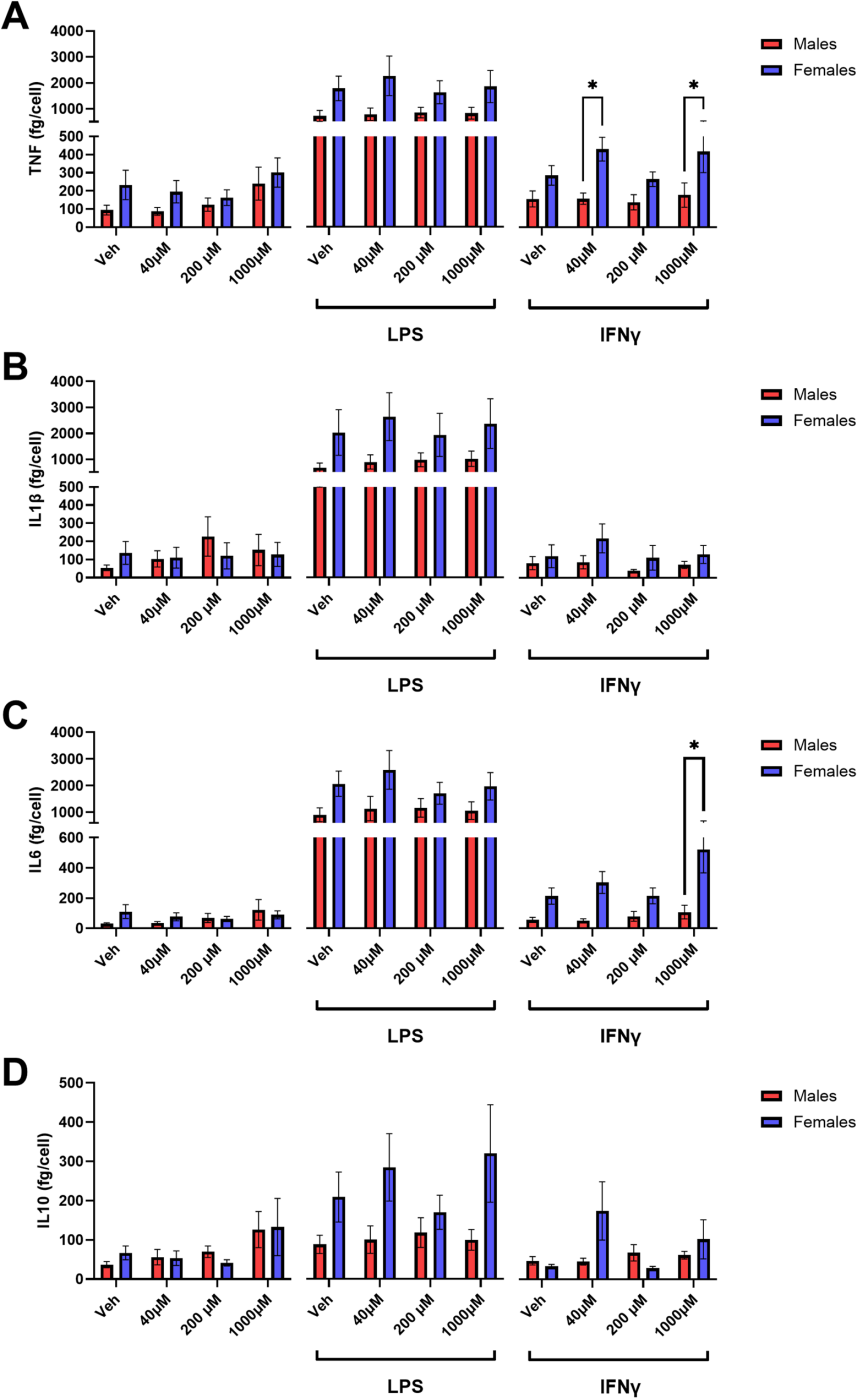
Microglia release of cytokines (**A**) TNF, (**B**) IL1β, (**C**) IL6, and (**D**) IL10 when treated with butyrate. Two-factor ANOVA identified a significant main effect of sex in all cytokines, and * indicates a pairwise statistical significance (*p* ≤ 0.05) between males (N = *7*) and females (N = *7*) with a two-factor ANOVA and Sidak’s multiple comparison *post hoc* (*N* = 14)

Given the observed sex differences in microglial morphology and response to butyrate in vivo, we next examined whether sex or butyrate treatment directly influenced microglial cytokine release in response to an inflammatory stimulus in vitro, without other indirect effects from cell signalling in the intact brain. Analysis of cytokines released after butyrate treatment and LPS or IFN stimulation revealed a significant main effect of sex but not butyrate treatment, with microglia isolated from female mice generally showing increased release of pro-inflammatory (IL6, TNF, IL1β) and anti-inflammatory (IL10) cytokines compared with males (Fig. 3). After stimulation with LPS or IFN, post-hoc analysis identified pairwise sex-dependent differences in pro-inflammatory cytokine release (IL6 and TNF; Fig. 3A, C). Of particular interest, females showed an increase in the IL6 response to IFNγ stimulation when treated with 1000 μm butyrate (Fig. 3I).

### Post-stroke butyrate supplementation does not improve stroke outcomes

**Fig. 4:**
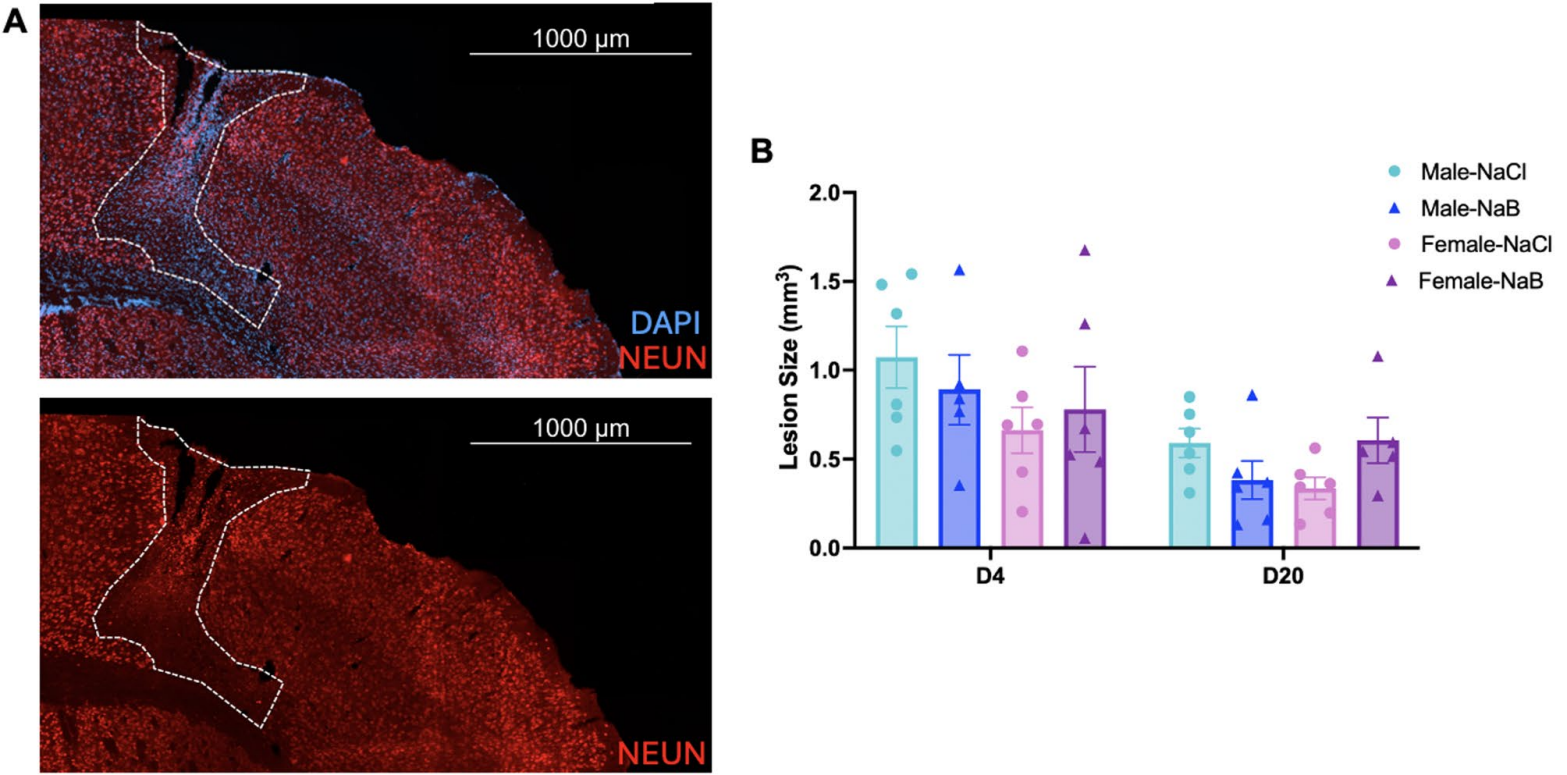
Butyrate treatment does not alter lesion volume. (**A**) Representative images of the stroke-injured cortex at D20. White dotted line depicts the lesioned area devoid of NEUN + neurons. (**B**) Lesion volume was measured in acute (D4) and chronic (D20) stroke. Measurements were performed on both male (blue) and female (pink/purple) animals that were treated with either vehicle (NaCl; light blue/pink) or butyrate (NaB; dark blue/purple). 3-way ANOVA with Tukey’s multiple comparisons test. Significant p values (< 0.05) are represented on graphs. N_D4−male−NaCl_ = 6 animals; N_D4−male−NaB_ = 5 animals; N_D4−female−NaCl_ = 3 animals; N_D4−female−NaB_ = 6 animals; N_D20−male−NaCl_ = 6 animals, N_D20−male−NaB_ = 6 animals; N_D20−female−NaCl_ = 6 animals; N_D20−female−NaB_ = 5 animals

**Fig. 5:**
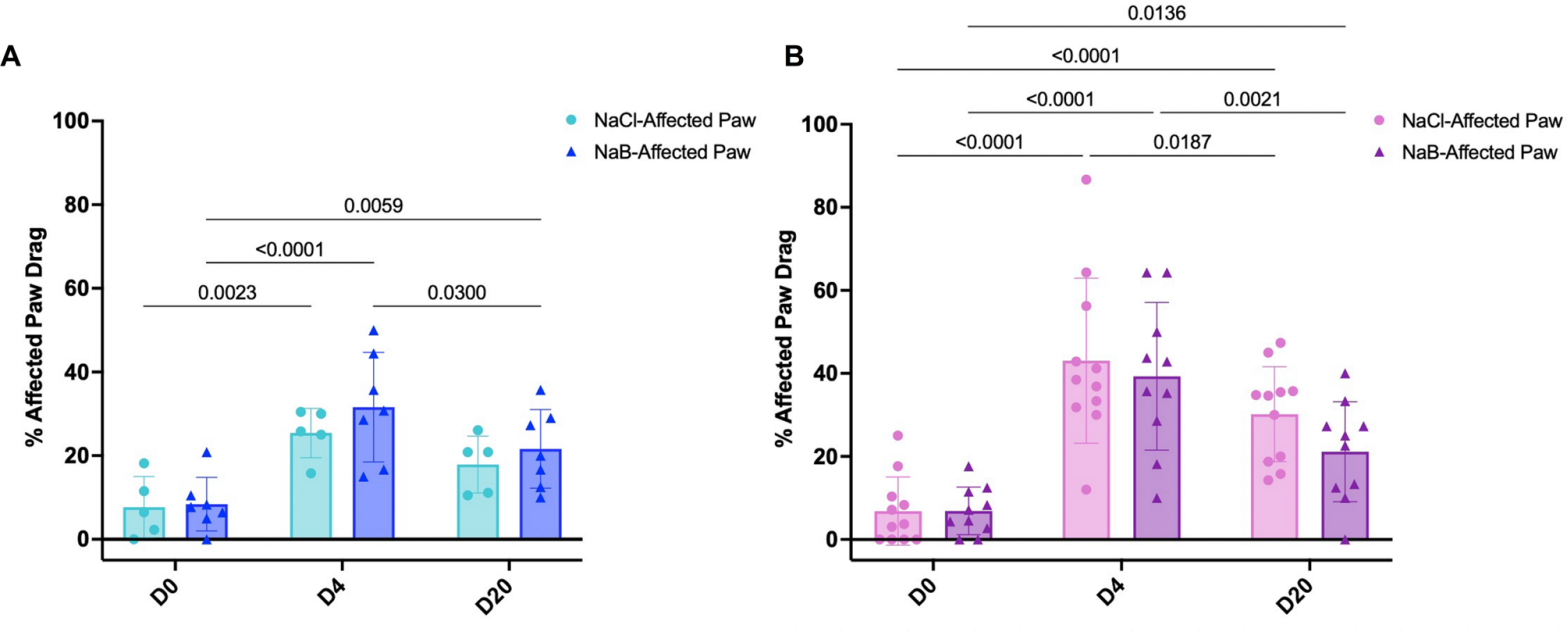
Post-stroke butyrate treatment does not lead to functional improvement. Paw dragging behaviour before stroke (D0), 4 days post-stroke (D4) and 20 days post-stroke (D20) in vehicle-treated (NaCl; light blue/pink) and butyrate-treated (NaB; dark blue/purple) male (**A**) and female (**B**) mice. 2-way ANOVA with uncorrected Fisher’s LSD. N_NaCl−Male_ = 5, N_NaB−Male_ = 7, N_NaCl−Female_ = 11, N_NaB−Female_ = 10

Given the observed effects on microglial morphology and cytokine release, we examined the effect of post-stroke butyrate treatment on stroke outcomes. When we examined lesion volume, no difference was observed between butyrate- and vehicle-treated animals at either D4 or D20, regardless of sex (Fig. 4). While stroke induction was sufficient to induce a motor deficit (Fig. 5, Fig S2), analysis of paw-dragging behaviour in the cylinder task showed no difference between treatment groups at D4 or D20 in both males (Fig. 5A, Additional File 2) and females (Fig. 5B, Additional File 2). These findings indicate that while post-stroke butyrate treatment induces sex-specific changes in microglial morphology and function, these changes alone are insufficient to improve outcomes with post-stroke treatment.

## Discussion

**Fig. 6:**
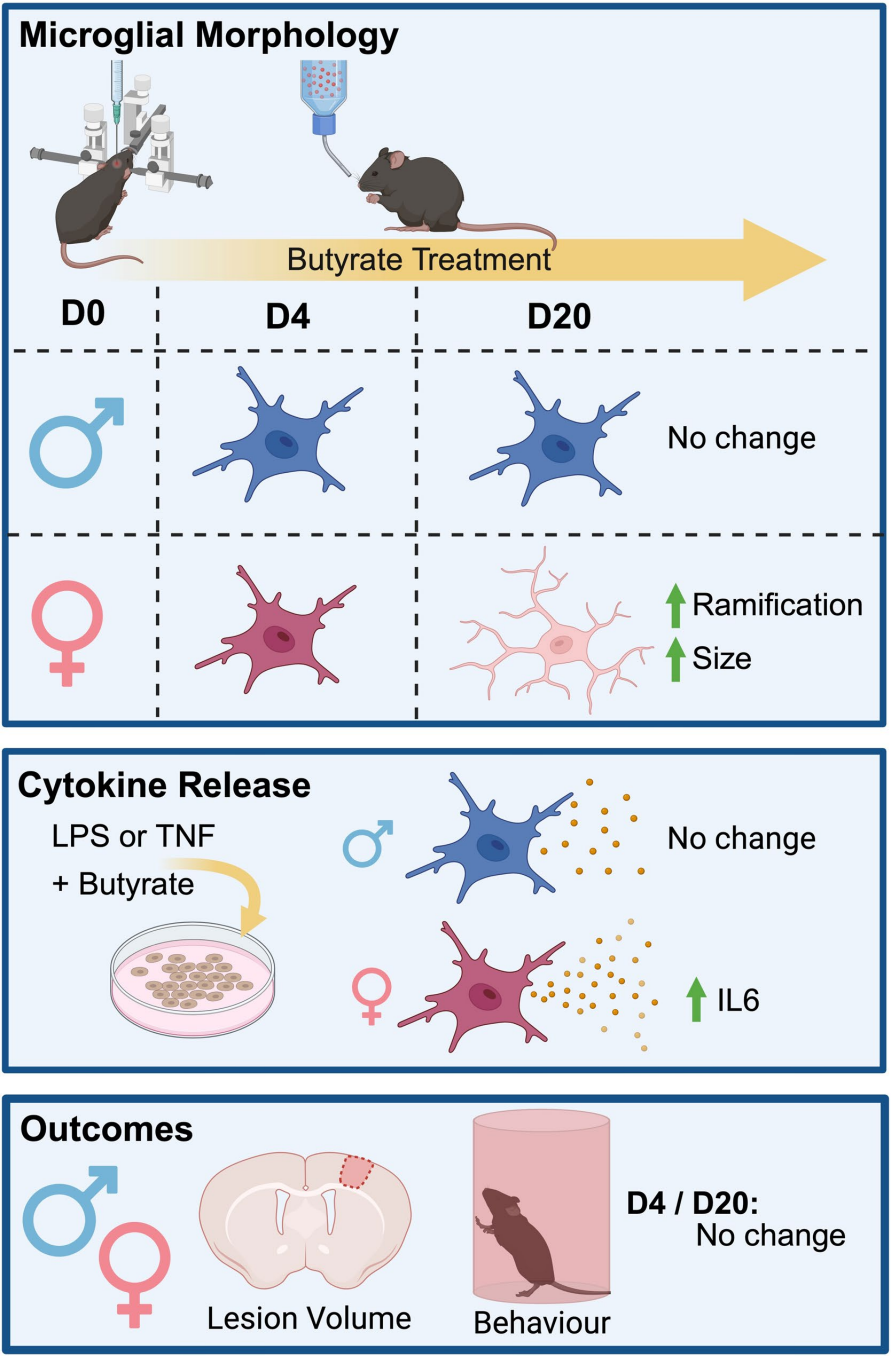
Schematic summary of experimental results. Post-stroke butyrate treatment increased microglial ramification and size at D20 post-stroke in females but had no effect in males. In vitro analysis revealed that butyrate enhanced IL6 release from microglia following IFNγ stimulation. Despite these microglial changes, post-stroke butyrate supplementation did not improve functional stroke outcomes in either sex, as assessed by lesion volume and cylinder task performance. Created with BioRender.com/6tfgzaz

The gut-brain axis has emerged as an exciting therapeutic target for stroke given that gut dysbiosis is associated with stroke prognosis [3]. One consequence of post-stroke dysbiosis is a reduction in the microbial production of SCCA metabolites that can signal to the CNS [4, 5]. Previous work has shown that supplementation with exogenous SCCAs can improve functional outcomes [6, 9, 18], with one recent study suggesting this occurs through alterations in microglial phenotypes [6]. However, the effect of individual SCCAs on microglial responses remained unclear, particularly across sexes and using a post-stroke treatment protocol. Therefore, in this study, we examined whether post-stroke supplementation with butyrate alone was sufficient to alter microglial function and improve stroke outcomes in male and female mice over time (Fig. 6).

Given that previous studies have suggested that SCCAs modulate microglia [6, 16, 30, 31], we began by assessing the impact of exogenous butyrate on the number and morphology of microglia post-stroke. We found no differences in the number of microglia in males or females treated with butyrate compared to vehicle controls at either D4 or D20 post-stroke. When we examined morphology, there was no change in microglial morphology following butyrate treatment in males at either timepoint or in females at D4. In contrast, at D20, butyrate-treated females showed an increase in microglial ramification.

We also noted baseline sex-differences in both microglial morphology and microglial cytokine release. Our findings are in line with previous studies showing sex-dependent changes in morphology in response to injury [32, 33]. Moreover, they support a previous study showing elevated expression of inflammatory mediators in female neonatal rats [34] and expand those findings by demonstrating that sex differences persist in the absence of hormones.

Despite the cellular changes in microglia, post-stroke butyrate treatment was insufficient to improve stroke outcomes as measured by lesion volume and sensorimotor behaviour. Our findings contrast with previous studies showing improved functional recovery following SCCA or butyrate supplementation [6, 9, 18]. This discrepancy may result from methodological differences in the timing of supplementation, dosing, route of administration, and formulations [6, 9, 18]. For example, Park and Sohrabji [18] used intraperitoneal injection of butyrate post-stroke in female rats and showed functional recovery. Chen et al. [9] administered butyrate intragastrically for two weeks post-stroke in male rats and reported improved neurological scores. Finally, Sadler et al. [6], used pre-supplementation of a SCCA cocktail (sodium propionate, sodium butyrate, and sodium acetate) in the drinking water and showed improvement in post-stroke motor deficits. While the butyrate dosage used in this study (40 mM) matches that of previous SCCA cocktail treatments [6, 35–39], some studies deliver up to 100–200 mM when supplementing a single SCCA such as acetate [40, 41] or butyrate [42–44]. These variations in administration and dosage suggest that differences in the bioavailability of SCCAs may play a role in treatment efficacy. The success of pre-supplementation paradigms indicates that propionic acid, acetic acid, and butyric acid may need to act synergistically for optimal recovery, or that pre-stroke gut microbiome composition influences treatment effectiveness [6].

This study is the first to compare the effect of butyrate on stroke across males and females. We identified sex differences in microglial morphology that align with previous findings in cortical brain injury, where males showed more ramified microglia than females post-injury [32]. These differences may reflect distinct developmental trajectories. Male microglial development stabilizes by P60 while female development continues [45], resulting in a more mature phenotype. This developmental divergence could result in different baseline phenotypes that could influence the response to butyrate treatment. The observed sex specific responses to butyrate treatment may also be explained by known sex differences in post-stroke gut dysbiosis. Females show higher SCCA levels than males following stroke [46, 47], which may contribute to these differential responses. These findings suggest that sex-specific dosing strategies may be warranted, potentially requiring higher doses in males to compensate for their baseline lower SCCA levels.

Several limitations should be considered when interpreting these findings. First, the use of young adult mice may not reflect the clinical population, as over half of ischemic strokes occur in humans over 70 years old [48]. Age has considerable impacts on stroke responses and outcomes, with older rodents experiencing greater functional impairment, larger lesions and worse recovery [49]. Additionally, since butyrate levels decline in older mice [50], butyrate supplementation may offer greater therapeutic benefit in aged animals than in younger ones. Another limitation of this study is the lack of controlled dosing. While oral administration is the most therapeutically relevant route, the drinking water delivery method used in this study prevents precise dosage control. Therefore, we recommend further investigation using micropipette-guided administration [51], which would provide accurate dosing while avoiding the stress associated with oral gavage. Finally, modelling the treatment effect of butyrate on isolated microglial cultures allowed us to directly test the hypothesis that butyrate acts via microglial modulation, however, this created additional limitations as this reductionist approach removes any potential influence of cell-cell interactions in an intact tissue microenvironment. Addressing sex differences in this isolated model was likewise limited by the absence of the intact tissue microenvironment and broader physiological influence on microglial responses, however, it did allow us to demonstrate differences in cellular response based only on the chromosomal differences between male and female-derived cells.

Nonetheless, our findings highlight several important considerations for future research. First, studies should examine various doses and administration routes for post-stroke butyrate treatment. Second, investigation of post-stroke supplementation with SCCA cocktails could determine whether combinatorial treatment proves more effective than single metabolites. Third, testing across different stroke models remains crucial, as our study using the endothelin-1 (ET-1) model differs from previous studies of photothrombotic or middle cerebral artery occlusion models [6, 9, 18, 52]. Finally, future work should examine the impact of post-stroke butyrate treatment across age groups and investigate transcriptional differences in microglia between sexes to better understand the mechanisms underlying sex-specific responses.

## Conclusions

This study investigated the effect of post-stroke butyrate supplementation across both sexes, revealing previously unknown sex differences in microglial responses to treatment (Fig. 6). Female microglia showed increased ramification at D20 and enhanced IL6 release following IFNγ stimulation in vitro, while males showed no microglial changes. Despite these novel microglial findings, butyrate treatment did not improve lesion volume or sensorimotor recovery in either sex. These results demonstrate the need to optimize post-stroke SCCA therapy for both sexes and highlight the importance of considering sex as a biological variable in developing stroke therapeutics.

## Supplementary Information

Additional file 1. Sholl analysis results. All raw Sholl analysis data and the averages for each animal sorted by condition and time point.

Additional file 2. Behavioural results. Raw behavioural data from the cylinder task for each animal, including animals excluded from the study.

Additional file 3. Supplementary Figure 1: Butyrate treatment eliminates sex differences in microglial morphology during chronic stroke. The number of primary branches was counted in acute (D4) (A) and chronic (D20) (B) stroke. The maximum number of intersections was measured in acute (C) and chronic (D) stroke. The radius at which the maximum number of intersections occurs was determined in acute (E) and chronic (F) stroke. All measurements were performed on both male and female animals, in addition to both butyrate-treated (blue and purple) and vehicle-treated (orange and green) animals. Each dot represents a cell. Each colour represents an animal. The horizontal dashed line represents the mean of the data. A 2-way ANOVA with uncorrected Fisher’s LSD was performed using the average cell morphology metrics per animal; p values (< 0.05) are represented on graphs. ND4-male-NaCl = 3 animals, 89 cells; ND4-male-NaB = 5 animals, 136 cells; ND4-female-NaCl = 4 animals, 111 cells; ND4-female-NaB = 6 animals, 186 cells; ND20-male-NaCl = 5 animals, 131 cells, ND20-male-NaB = 4 animals, 87 cells; ND20-female-NaCl = 5 animals, 113 cells; ND20-female-NaB = 5 animals, 117 cells.

Additional file 4. Supplemental Figure 2: A triple injection of endothelin-1 is sufficient to induce motor deficits. Left forelimb slippage (A) and left hindlimb slippage (B) was assessed before injury (D0) and 4 days post-injury (D4) in both sham-injured (green) and stroke-injured (blue) male mice. 2-way ANOVA with uncorrected Fisher’s LSD. Nsham = 6, Nstroke = 8.

## Data Availability

All data generated or analysed during this study are available from the corresponding author on reasonable request. The source data that support the findings in Figs. 2 and 5 is provided in Supplementary Tables 1 and 2.

## Abbreviations

SCCAs: Short-chain carboxylic acids
ET-1: Endothelin-1

## References

[1] Kuriakose D, Xiao Z. Pathophysiology and treatment of stroke: present status and future perspectives. Int J Mol Sci. 2020;21:7609. 33076218 10.3390/ijms21207609 PMC7589849

[2] Zhu H, Hu S, Li Y, Sun Y, Xiong X, Hu X, et al. Interleukins and ischemic stroke. Front Immunol. 2022;13:828447. 35173738 10.3389/fimmu.2022.828447 PMC8841354

[3] Peh A, O’Donnell JA, Broughton BRS, Marques FZ. Gut microbiota and their metabolites in stroke: A Double-Edged sword. Stroke. 2022;53:1788–801. 35135325 10.1161/STROKEAHA.121.036800

[4] Li H, Zhang X, Pan D, Liu Y, Yan X, Tang Y, et al. Dysbiosis characteristics of gut microbiota in cerebral infarction patients. Transl Neurosci. 2020;11:124–33. 33312718 10.1515/tnsci-2020-0117 PMC7706127

[5] Tan C, Wu Q, Wang H, Gao X, Xu R, Cui Z, et al. Dysbiosis of gut microbiota and Short-Chain fatty acids in acute ischemic stroke and the subsequent risk for poor functional outcomes. JPEN J Parenter Enter Nutr. 2021;45:518–29. 10.1002/jpen.1861 PMC8048557 32473086

[6] Sadler R, Cramer JV, Heindl S, Kostidis S, Betz D, Zuurbier KR, et al. Short-Chain fatty acids improve poststroke recovery via immunological mechanisms. J Neurosci. 2020;40:1162–73. 31889008 10.1523/JNEUROSCI.1359-19.2019 PMC6989004

[7] Frost G, Sleeth ML, Sahuri-Arisoylu M, Lizarbe B, Cerdan S, Brody L, et al. The short-chain fatty acid acetate reduces appetite via a central homeostatic mechanism. Nat Commun. 2014;5:3611. 24781306 10.1038/ncomms4611 PMC4015327

[8] Morrison DJ, Preston T. Formation of short chain fatty acids by the gut microbiota and their impact on human metabolism. Gut Microbes. 2016;7:189–200. 26963409 10.1080/19490976.2015.1134082 PMC4939913

[9] Chen R, Xu Y, Wu P, Zhou H, Lasanajak Y, Fang Y, et al. Transplantation of fecal microbiota rich in short chain fatty acids and Butyric acid treat cerebral ischemic stroke by regulating gut microbiota. Pharmacol Res. 2019;148:104403. 31425750 10.1016/j.phrs.2019.104403

[10] Tay TL, Savage JC, Hui CW, Bisht K, Tremblay M-È. Microglia across the lifespan: from origin to function in brain development, plasticity and cognition. J Phys. 2016;595:1929–45. 10.1113/JP272134 PMC5350449 27104646

[11] Guo S, Wang H, Yin Y. Microglia polarization from M1 to M2 in neurodegenerative diseases. Front Aging Neurosci. 2022;14:815347. 35250543 10.3389/fnagi.2022.815347 PMC8888930

[12] Wang J, Xing H, Wan L, Jiang X, Wang C, Wu Y. Treatment targets for M2 microglia polarization in ischemic stroke. Biomed Pharmacother. 2018;105:518–25. 29883947 10.1016/j.biopha.2018.05.143

[13] Fan P, Wang S, Chu S, Chen N. Time-dependent dual effect of microglia in ischemic stroke. Neurochem Int. 2023;169:105584. 37454817 10.1016/j.neuint.2023.105584

[14] Pawluk H, Woźniak A, Grześk G, Kołodziejska R, Kozakiewicz M, Kopkowska E, et al. The role of selected Pro-Inflammatory cytokines in pathogenesis of ischemic stroke. Clin Interv Aging. 2020;15:469–84. 32273689 10.2147/CIA.S233909 PMC7110925

[15] Yenari MA, Kauppinen TM, Swanson RA. Microglial activation in stroke: therapeutic targets. Neurotherapeutics. 2010;7:378–91. 20880502 10.1016/j.nurt.2010.07.005 PMC5084300

[16] Caetano-Silva ME, Rund L, Hutchinson NT, Woods JA, Steelman AJ, Johnson RW. Inhibition of inflammatory microglia by dietary fiber and short-chain fatty acids. Sci Rep. 2023;13:2819. 36797287 10.1038/s41598-022-27086-x PMC9935636

[17] Churchward MA, Michaud ER, Mullish BH, Miguens Blanco J, Garcia Perez I, Marchesi JR, et al. Short-chain fatty and carboxylic acid changes associated with fecal microbiota transplant communally influence microglial inflammation. Heliyon. 2023;9:e16908. 37484415 10.1016/j.heliyon.2023.e16908 PMC10360965

[18] Park MJ, Sohrabji F. The histone deacetylase inhibitor, sodium butyrate, exhibits neuroprotective effects for ischemic stroke in middle-aged female rats. J Neuroinflamm. 2016;13:300. 10.1186/s12974-016-0765-6 PMC5131416 27905989

[19] Lapchak PA, Zhang JH, Noble-Haeusslein LJ. RIGOR guidelines: escalating STAIR and STEPS for effective translational research. Transl Stroke Res. 2013;4:279–85. 23658596 10.1007/s12975-012-0209-2 PMC3644408

[20] Daniele E, Nazer Y, Kortebi I, Casasbuenas DL, Fan Y, Trinh M, et al. Oral probiotic therapy improves motor function in a rodent model of sensorimotor stroke. Exp Brain Res. 2023;241:1931–43. 37358570 10.1007/s00221-023-06651-4

[21] Metz GA, Whishaw IQ. the ladder rung walking task: a scoring system and its practical application. J Vis Exp JoVE. 2009; 28:1204 10.3791/1204 PMC2796662 19525918

[22] Schindelin J, Arganda-Carreras I, Frise E, Kaynig V, Longair M, Pietzsch T, et al. Fiji: an open-source platform for biological-image analysis. Nat Methods. 2012;9:676–82. 22743772 10.1038/nmeth.2019 PMC3855844

[23] Ferreira TA, Blackman AV, Oyrer J, Jayabal S, Chung AJ, Watt AJ, et al. Neuronal morphometry directly from bitmap images. Nat Methods. 2014;11:982–4. 25264773 10.1038/nmeth.3125 PMC5271921

[24] Sholl DA. Dendritic organization in the neurons of the visual and motor cortices of the Cat. J Anat. 1953;87:387–406. 13117757 PMC1244622

[25] Arshadi C, Günther U, Eddison M, Harrington KIS, Ferreira TA. SNT: a unifying toolbox for quantification of neuronal anatomy. Nat Methods. 2021;18:374–7. 33795878 10.1038/s41592-021-01105-7

[26] Schneider CA, Rasband WS, Eliceiri KW. NIH image to imageJ: 25 years of image analysis. Nat Methods. 2012;9:671–5. 22930834 10.1038/nmeth.2089 PMC5554542

[27] Bankhead P, Loughrey MB, Fernández JA, Dombrowski Y, McArt DG, Dunne PD, et al. QuPath: open source software for digital pathology image analysis. Sci Rep. 2017;7:16878. 29203879 10.1038/s41598-017-17204-5 PMC5715110

[28] Saura J, Tusell JM, Serratosa J. High-yield isolation of murine microglia by mild trypsinization. Glia. 2003;44:183–9. 14603460 10.1002/glia.10274

[29] Sun J, Ling Z, Wang F, Chen W, Li H, Jin J, et al. Clostridium butyricum pretreatment attenuates cerebral ischemia/reperfusion injury in mice via anti-oxidation and anti-apoptosis. Neurosci Lett. 2016;613:30–5. 26733300 10.1016/j.neulet.2015.12.047

[30] Ziabska K, Gargas J, Sypecka J, Ziemka-Nalecz M. The impact of the histone deacetylase inhibitor sodium butyrate on microglial polarization after oxygen and glucose deprivation. Pharmacol Rep. 2022;74:909–19. 35796871 10.1007/s43440-022-00384-x

[31] Wenzel TJ, Gates EJ, Ranger AL, Klegeris A. Short-chain fatty acids (SCFAs) alone or in combination regulate select immune functions of microglia-like cells. Mol Cell Neurosci. 2020;105:103493. 32333962 10.1016/j.mcn.2020.103493

[32] Acaz-Fonseca E, Duran JC, Carrero P, Garcia-Segura LM, Arevalo MA. Sex differences in glia reactivity after cortical brain injury. Glia. 2015;63:1966–81. 26037411 10.1002/glia.22867

[33] Villapol S, Loane DJ, Burns MP. Sexual dimorphism in the inflammatory response to traumatic brain injury. Glia. 2017;65:1423–38. 28608978 10.1002/glia.23171 PMC5609840

[34] Schwarz JM, Sholar PW, Bilbo SD. Sex differences in microglial colonization of the developing rat brain. J Neurochem. 2012;120:948–63. 22182318 10.1111/j.1471-4159.2011.07630.x PMC3296888

[35] Kaye DM, Shihata WA, Jama HA, Tsyganov K, Ziemann M, Kiriazis H, et al. Deficiency of prebiotic Fiber and insufficient signaling through gut Metabolite-Sensing receptors leads to cardiovascular disease. Circulation. 2020;141:1393–403. 32093510 10.1161/CIRCULATIONAHA.119.043081

[36] Lee JG, Lee J, Lee A, Jo SV, Park CH, Han DS, et al. Impact of short-chain fatty acid supplementation on gut inflammation and microbiota composition in a murine colitis model. J Nutr Biochem. 2022;101:108926. 34848335 10.1016/j.jnutbio.2021.108926

[37] van de Wouw M, Boehme M, Lyte JM, Wiley N, Strain C, O’Sullivan O, et al. Short-chain fatty acids: microbial metabolites that alleviate stress‐induced brain–gut axis alterations. J Physiol. 2018;596:4923–44. 30066368 10.1113/JP276431 PMC6187046

[38] Smith PM, Howitt MR, Panikov N, Michaud M, Gallini CA, Bohlooly-Y M et al (2013) The microbial metabolites, short chain fatty acids, regulate colonic Treg cell homeostasis. Science 341. 10.1126/science.1241165 23828891 10.1126/science.1241165 PMC3807819

[39] Erny D, Hrabě de Angelis AL, Prinz M. Communicating systems in the body: how microbiota and microglia cooperate. Immunology. 2017;150:7–15. 27392533 10.1111/imm.12645 PMC5341501

[40] Marques FZ, Nelson E, Chu P-Y, Horlock D, Fiedler A, Ziemann M, et al. High-Fiber diet and acetate supplementation change the gut microbiota and prevent the development of hypertension and heart failure in hypertensive mice. Circulation. 2017;135:964–77. 27927713 10.1161/CIRCULATIONAHA.116.024545

[41] Thorburn AN, McKenzie CI, Shen S, Stanley D, Macia L, Mason LJ, et al. Evidence that asthma is a developmental origin disease influenced by maternal diet and bacterial metabolites. Nat Commun. 2015;6:7320. 26102221 10.1038/ncomms8320

[42] Fachi JL, Felipe J, de Pral S, Silva LP, Corrêa BK, de Andrade RO et al (2019) Butyrate Protects Mice from Clostridium difficile-Induced Colitis through an HIF-1-Dependent Mechanism. Cell Reports 27:750–761 30995474 10.1016/j.celrep.2019.03.054

[43] Xiao P, Cai X, Zhang Z, Guo K, Ke Y, Hu Z, et al. Butyrate prevents the pathogenic Anemia-Inflammation circuit by facilitating macrophage Iron export. Adv Sci. 2024;11:2306571. 10.1002/advs.202306571 PMC10966513 38235606

[44] Fang W, Xue H, Chen X, Chen K, Ling W. Supplementation with sodium butyrate modulates the composition of the gut microbiota and ameliorates High-Fat Diet-Induced obesity in mice. J Nutr. 2019;149:747–54. 31004166 10.1093/jn/nxy324

[45] Hanamsagar R, Alter MD, Block CS, Sullivan H, Bolton JL, Bilbo SD. Generation of a microglial developmental index in mice and in humans reveals a sex difference in maturation and immune reactivity. Glia. 2017;65:1504–20. 28618077 10.1002/glia.23176 PMC5540146

[46] Wang J, Zhong Y, Zhu H, Mahgoub OK, Jian Z, Gu L, et al. Different gender-derived gut microbiota influence stroke outcomes by mitigating inflammation. J Neuroinflamm. 2022;19:245. 10.1186/s12974-022-02606-8 PMC9531521 36195899

[47] El-Hakim Y, Mani KK, Eldouh A, Pandey S, Grimaldo MT, Dabney A, et al. Sex differences in stroke outcome correspond to rapid and severe changes in gut permeability in adult Sprague-Dawley rats. Biol Sex Differ. 2021;12:14. 33451354 10.1186/s13293-020-00352-1 PMC7811247

[48] Feigin VL, Brainin M, Norrving B, Martins SO, Pandian J, Lindsay P, et al. World stroke organization: global stroke fact sheet 2025. Int J Stroke. 2025;20:132. 39635884 10.1177/17474930241308142 PMC11786524

[49] Zhang H, Lin S, Chen X, Gu L, Zhu X, Zhang Y, et al. The effect of age, sex and strains on the performance and outcome in animal models of stroke. Neurochem Int. 2019;127:2–11. 30291954 10.1016/j.neuint.2018.10.005

[50] Lee J, d’Aigle J, Atadja L, Quaicoe V, Honarpisheh P, Ganesh BP, et al. Gut Microbiota-Derived Short-Chain fatty acids promote poststroke recovery in aged mice. Circ Res. 2020;127:453–65. 32354259 10.1161/CIRCRESAHA.119.316448 PMC7415518

[51] Heraudeau M, Roux CM, Lahogue C, Largilliere S, Allouche S, Lelong-Boulouard V, et al. Micropipette-guided drug administration (MDA) as a non-invasive chronic oral administration method in male rats. J Neurosci Methods. 2023;398:109951. 37634649 10.1016/j.jneumeth.2023.109951

[52] Sommer CJ. Ischemic stroke: experimental models and reality. Acta Neuropathol. 2017;133:245–61. 28064357 10.1007/s00401-017-1667-0 PMC5250659

