# Supplementary figures and images for: Post-stroke butyrate treatment shows sex-dependent microglial responses but does not improve outcomes in a mouse model of endothelin-1 sensory motor stroke
Source: BMC Neurosci. 2025 Jul 17;26:43. doi: 10.1186/s12868-025-00959-3 (PMC12273294; doi:10.1186/s12868-025-00959-3)

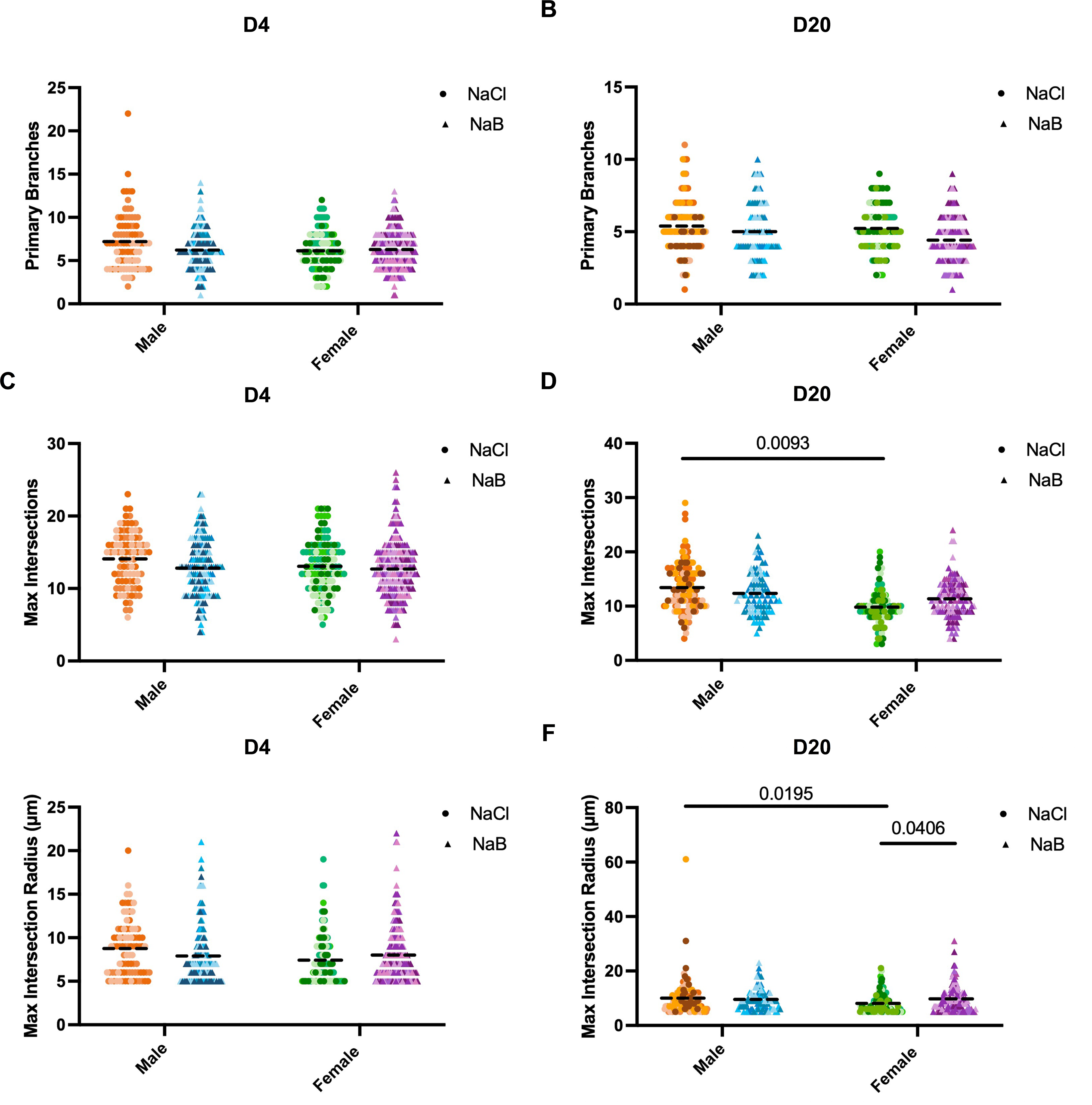

Supplement: Supplementary file 3 — Additional file 3. Supplementary Figure 1: Butyrate treatment eliminates sex differences in microglial morphology during chronic stroke. The number of primary branches was counted in acute (D4) (A) and chronic (D20) (B) stroke. The maximum number of intersections was measured in acute (C) and chronic (D) stroke. The radius at which the maximum number of intersections occurs was determined in acute (E) and chronic (F) stroke. All measurements were performed on both male and female animals, in addition to both butyrate-treated (blue and purple) and vehicle-treated (orange and green) animals. Each dot represents a cell. Each colour represents an animal. The horizontal dashed line represents the mean of the data. A 2-way ANOVA with uncorrected Fisher’s LSD was performed using the average cell morphology metrics per animal; p values (< 0.05) are represented on graphs. ND4-male-NaCl = 3 animals, 89 cells; ND4-male-NaB = 5 animals, 136 cells; ND4-female-NaCl = 4 animals, 111 cells; ND4-female-NaB = 6 animals, 186 cells; ND20-male-NaCl = 5 animals, 131 cells, ND20-male-NaB = 4 animals, 87 cells; ND20-female-NaCl = 5 animals, 113 cells; ND20-female-NaB = 5 animals, 117 cells. [file 12868_2025_959_MOESM3_ESM.tif]

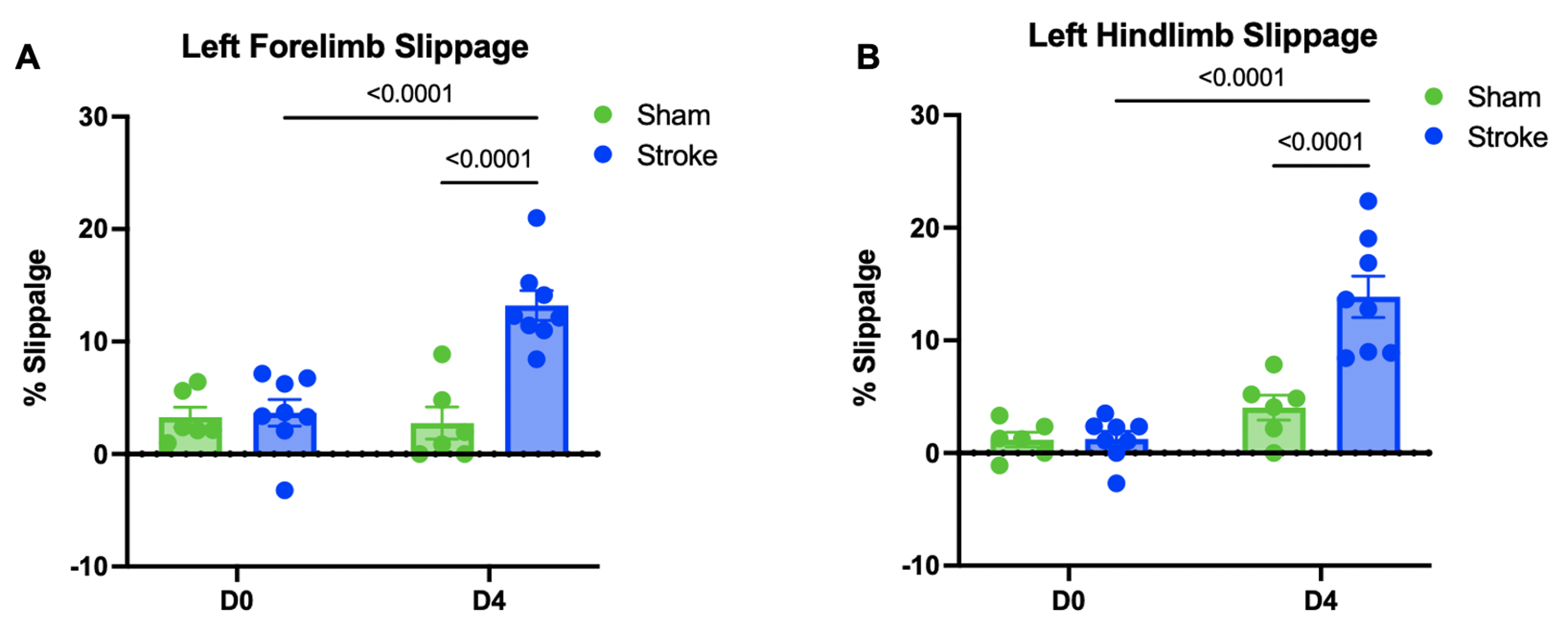

Supplement: Supplementary file 4 — Additional file 4. Supplemental Figure 2: A triple injection of endothelin-1 is sufficient to induce motor deficits. Left forelimb slippage (A) and left hindlimb slippage (B) was assessed before injury (D0) and 4 days post-injury (D4) in both sham-injured (green) and stroke-injured (blue) male mice. 2-way ANOVA with uncorrected Fisher’s LSD. Nsham = 6, Nstroke = 8. [file 12868_2025_959_MOESM4_ESM.tif]
